# Characteristics of human papillomaviruses distribution in Guizhou Province, China

**DOI:** 10.1186/s12985-019-1239-0

**Published:** 2019-10-29

**Authors:** Zuyi Chen, Qiongyao Li, Qiong Huang, Huaqing Liu, Hongwu Jiang, Zehui Chen, Zhengyuan An, Qingfang Luo

**Affiliations:** 1 Department of Laboratory Medicine, Affiliated Hospital of Zunyi Medical University, Zunyi, Guizhou People’s Republic of China; 2 Department of Information Technology, Affiliated Hospital of Zunyi Medical University, Zunyi, Guizhou People’s Republic of China; 3 Department of Pathology, Affiliated Hospital of Zunyi Medical University, Zunyi, Guizhou People’s Republic of China; 4 School of Laboratory Medicine, Zunyi Medical University, Zunyi, Guizhou People’s Republic of China

**Keywords:** Human papillomavirus, Season, Age group, Distribution

## Abstract

**Background:**

Human papillomavirus (HPV) is one of the most common sexually transmitted viruses. Data about HPV infection in Guizhou is limited.

**Methods:**

56,768 cervical samples were collected and genotyped for 15 main high risk and 6 main low risk HPV types.

**Results:**

16.95% (9623/56768) of samples were HPV positive; 90.70% (8728/9623) of HPV positive women were infected by high risk HPV. High risk and high risk mix infection (1458; 70.85%) was the most common mix HPV infection type. The highest HPV detection rate was found in age group 41–45 years old (detection rate = 17.89%) (χ2 = 204.77; *P* < 0.001); the highest within-group HPV infection rates were found in the ≤20 (25.62%) and ≥ 61 (24.67%) years old age groups, the lowest within-group HPV infection rate was found in the 31–35 years old age group (15.02%). The highest mix infection proportions were found in the ≥61 (36.06%) and ≤ 20 (33.63%) years old age groups (χ2 = 111.21; *P* < 0.001), the lowest mix infection proportion was found in the 41–45 (17.42%) years old age group. The highest high risk infection proportions were found in the 26–30 (92.98%), ≥61 (92.68%), and 36–40 (92.16%) years old age groups (χ2 = 31.72; *P* < 0.001), the lowest high risk infection proportion was found in the ≤20 (84.96%) years old age group. HPV infection rates varied with seasons in Guizhou.

**Conclusions:**

Characteristics of HPV distribution in Guizhou were identified. There were significant differences in HPV distribution among age groups, prevention strategies should be adjusted according to the characteristics.

## Introduction

Human papillomavirus (HPV) is associated with varied epithelial lesions, including cervical intraepithelial neoplasia and genital warts [1]. Cervical cancer is one of the most common malignant tumors among women worldwide, genital warts is also very prevalent among women; each year about 500,000 new cases of cervical cancer and 1 million new cases of genital warts were diagnosed worldwide [2, 3].

More than 200 HPV types have been identified until now (pave.niaid.nih.gov). “High-risk” and “low-risk” were used to distinguish different HPV types according to their risks in causing malignant tumors. HPV 16, 18, 31, 33, 35, 39, 45, 51, 52, 53, 56, 58, 59, 66, 68, 73, and 83 are common high risk types; HPV 6, 11, 40, 42, 43, 44, 54, 61, 70, 72, and 81 are common low risk types [4].

HPV distribution varies geographically considerably. Effective HPV prevention and treatment strategies should be specialized based on local HPV distribution characteristics [5]. There is little data about HPV distribution characteristics in Guizhou, China. Guizhou is a province with 36 million people (data from National Bureau of Statistics of China, http://www.stats.gov.cn/). This study aimed to investigate HPV distribution characteristics in Guizhou, China, to afford data for HPV prevention and treatment locally.

## Methods and materials

From January 1, 2016 to December 31, 2017, cervical swabs were obtained from patients at the Affiliated Hospital of Zunyi Medical University. Women over 18 years old with visible cervical lesions and/or HPV-related diseases (e.g. cervical intraepithelial neoplasia) were eligible for inclusion. The present study was approved by the Affiliated Hospital of Zunyi Medical University Ethics Committee, the approval number was ZYFYLS2018(81). Before sample collection, informed consent was obtained.

Cervical specimens were collected by cervical brush and stored in preservative buffer solution (NaCl 9 g, sodium benzoate 10 g, H_2_O 1 L) at − 20 °C. Cervical specimens were detected for HPV Genotyping by a Kit (Human Papillomavirus Genotyping Kit; Yaneng Bioscience, Shenzhen, China) according to the instruction. Positive and negative controls were included in all experiments.

SPSS version 19 (IBM, Armonk, NY, USA) was used for data analysis. The Pearson χ2 test was used to confirm the results. *P* < 0.05 was considered statistically significant.

## Results

In total, 56,768 samples (mean age 39.45 ± 9.88 years old) were collected and detected for HPV genotyping, 16.95% (9623/56768) of samples were HPV positive; 90.70% (8728/9623) of HPV positive patients were infected by high risk HPV. HPV detection number was 12,378, 87.66% (10,850/12378) were high-risk HPV types. Detection number and detection rate of high-risk types were HPV52 (2206; 17.82%), HPV16 (1713; 13.84%), HPV58 (1435; 11.59%), HPV53 (986; 7.97%), HPV39 (923; 7.46%), HPV51 (723; 5.84%), HPV18 (534; 4.31%), HPV33 (523; 4.23%), HPV68 (456; 3.68%), HPV31 (380; 3.07%), HPV66 (309; 2.50%), HPV56 (238; 1.92%), HPV59 (210; 1.70%), HPV45 (110; 0.89%), and HPV35 (104; 0.84%). Detection number and detection rate of low-risk types were HPV81 (769; 6.21%), HPV6 (262; 2.12%), HPV11 (241; 1.95%), HPV44 (145; 1.17%), HPV43 (63; 0.51%), and HPV42 (48; 0.39%).

Women were divided into different age groups. The highest HPV detection rate was found in age group 41–45 years old (detection rate = 17.89%) (χ2 = 204.77; *P* < 0.001) (see Table 1); but the highest within-group HPV infection rates were found in the ≤20 (25.62%) and ≥ 61 (24.67%) years old age groups, the lowest within-group HPV infection rate was found in the 31–35 years old age group (15.02%) (see Table 1). The highest mix infection proportions were found in the ≥61 (36.06%) and ≤ 20 (33.63%) years old groups (χ2 = 111.21; *P* < 0.001), the lowest mix infection proportion was found in the 41–45 (17.42%) years old age group (see Table 2). The highest high risk infection proportions were found in the 26–30 (92.98%), ≥61 (92.68%), and 36–40 (92.16%) years old groups (χ2 = 31.72; *P* < 0.001), the lowest high risk infection proportion was found in the ≤20 (84.96%) years old age group (see Table 2).

**Table 1:** Detection rate and positive rate of patients in each age group

age	Positive number	Negative number	Total	Detection rate	Positive rate
≤20	113	328	441	1.17%	25.62%
21–25	739	2807	3546	7.68%	20.84%
26–30	1410	6765	8175	14.65%	17.25%
31–35	1285	7273	8558	13.35%	15.02%
36–40	1530	8280	9810	15.90%	15.60%
41–45	1722	9331	11,053	17.89%	15.58%
46–50	1320	6469	7789	13.72%	16.95%
51–55	871	3821	4692	9.05%	18.56%
56–60	278	987	1265	2.89%	21.98%
≥61	355	1084	1439	3.69%	24.67%
Total	9623	47,145	56,768	100.00%	16.95%

Note: Detection rate means positive number percentage of each age group in total positive number. After rounding of “Detection rate” in each age group, the “Detection rate” arithmetic sum of all age group was not 100.00%

**Table 2:** High risk proportion and mix infection proportion of each age group

age	High risk infection	Only low risk infection	High risk proportion	Mix infection	Single infection	Mix infection proportion
≤20	96	17	84.96%	38	75	33.63%
21–25	676	63	91.47%	193	546	26.12%
26–30	1311	99	92.98%	313	1097	22.20%
31–35	1168	117	90.89%	249	1036	19.38%
36–40	1410	120	92.16%	272	1258	17.78%
41–45	1527	195	88.68%	300	1422	17.42%
46–50	1183	137	89.62%	299	1021	22.65%
51–55	777	94	89.21%	182	689	20.90%
56–60	251	27	90.29%	84	194	30.22%
≥61	329	26	92.68%	128	227	36.06%
total	8728	895	90.70%	2058	7565	21.39%

Note: High risk infection, Only low risk infection, Mix infection, Single infection mean positive patients number of high risk infection, only low risk infection, mix infection, and single infection, respectively. High risk proportion means high risk infection number percentage of each age group in total number of both high risk infection and only low risk infection. Mix infection proportion means mix infection number percentage of each age group in total number of both mix infection and single infection

The number and detection rate of different HPV types detected per patient were listed in Table 3. At most eight infection was found. High risk and high risk mix infection (1458; 70.85%) was the most common mix HPV infection type with overriding advantage (see Table 3).

**Table 3:** Percentage of single infection and mixed infections

Type	Number	Positive rate	Detection rate
single	7565	13.326%	78.61%
double	1561	2.750%	16.22%
three	360	0.634%	3.74%
four	97	0.171%	1.01%
five	25	0.044%	0.26%
six	8	0.014%	0.08%
seven	6	0.011%	0.06%
eight	1	0.002%	0.01%
Total	9623	16.95%	100.00%
HR-HR	1458	2.57%	70.85%
HR-LR	580	1.02%	28.18%
LR-LR	20	0.04%	0.97%
Total	2058	3.63%	100.00%

Note: Single, double, three, four, five, six, seven, eight means single, double, three, four, five, six, seven, eight infection, respectively. HR-HR means high-risk and high-risk mixed infection; HR-LR means high-risk and low-risk mixed infection; LR-LR means low-risk and low-risk mixed infection. Positive rate means positive number percentage of each infection type in total patients. Detection rate means positive number percentage of each infection type in total positive number. After rounding, the “Detection rate” arithmetic sum of single, double, three, four, five, six, seven and eight infection was not 100.00%

HPV positive rates of different seasons were analyzed. Months were divided into Spring (March, April and May), Summer (June, July and August), Autumn (September, October and November) and Winter (January, February and December) according to the standard QX/T152–2012 of China Meteorological Information Center (data.cma.cn). We found that positive rate of HPV varied with seasons. The highest and lowest (χ2 = 39.46.; *P* < 0.001) HPV positive rates were found in Spring (2699/14600; 18.49%) and Autumn (2148/13601; 15.79%), respectively (see Table 4).

**Table 4:** Positive rates of four seasons

Season	Positive number	Negative number	Positive rate
Spring	2699	11,901	18.49%
Summer	2848	14,369	16.54%
Autumn	2148	11,453	15.79%
Winter	1928	9422	16.99%
Total	9623	47,145	16.95%

Note: Positive number and Negative number mean HPV positive and HPV negative number patients in each season. Positive rate means positive number percentage in total patients of each season

## Discussion

This study investigated the HPV distribution characteristics in Guizhou. This is the first HPV distribution study with large sample size in Guizhou. Data presented in this study was useful for HPV detection, prevention and treatment in Guizhou.

HPV16, HPV18, HPV31, HPV58 and HPV52 were the five most common high risk HPV types worldwide; HPV16 and HPV18 account for approximately 70% of cervical cancer cases [6, 7]. While HPV16, HPV52, HPV18, HPV51 and HPV58 were the five most common high risk HPV types in China [6]. In the present study, HPV52, HPV16, HPV58, HPV53 and HPV39 were the five most common high risk HPV types in Guizhou. HPV53, HPV39 and HPV51 were more common than HPV18; HPV53 and HPV51 were rarely reported as the most common types in China recently [8–14]. No HPV vaccine target HPV53, HPV39 and HPV51 until now. So HPV53, HPV39 and HPV51 should be important targets of high risk HPV prevention by detection. HPV vaccine for HPV53, HPV39 and HPV51 should be developed for Guizhou.

HPV6 and HPV11 were the most common low risk HPV types worldwide and in China [9]. But in the present study, we found that HPV81 was the most common low risk type with overwhelming advantage. And what was worse, no HPV vaccine target HPV81 until now. So low risk HPV prevention key target by detection may should be adjusted from HPV6 and HPV11 to HPV81. HPV81 vaccine should be developed for Guizhou.

High-risk HPV persistent infection is the main reason of cervical cancer [15]. In the present study, we found that the high risk HPV infection proportions were up to 90.70 and 92.98% in all and in 26–30 years old age group HPV positive women, respectively. Proportion of high risk HPV infection in Guizhou was much higher than studies in other recent reports in China [8–14].

In the present study, we found that the most prevalent HPV infection age was between 41 and 45 years old (detection rate = 17.89%), similar to few recent studies in China [9, 10], but different from most studies in and out of China [8, 11–14, 16, 17]. The HPV with-in group positive rate, mix infection proportion and high risk infection proportion were all relatively high in age group ≥61. Ideal prevention strategies for Guizhou population should be developed according to the HPV distribution characteristics of each age group in Guizhou, especially for the age groups with highest HPV with-in group positive rates, mix infection proportions and high risk infection proportions.

Whether HPV mix infections increase the risk of disease is not clear. Antigens vary among different HPV types; cross-defense is not effective enough [18]. HPV mix infection may increase the risk of HPV-related diseases. Compared to similar studies, high risk and high risk HPV mix infection in Guizhou was very high [8, 9]. Age groups with higher HPV mix infection in the present study may need more attention in HPV prevention in Guizhou.

In this study, we found that there were statistical differences in HPV positive rates among different seasons, indicating that season factor should also be taken in to consideration in HPV prevention in Guizhou.

## Conclusion

HPV52, HPV16, HPV58, HPV53 and HPV39 were the five most common high risk HPV types in Guizhou. HPV81 was the most common low risk type in Guizhou. High risk HPV infection proportion was very high in Guizhou. There were significant differences in HPV distribution among age groups, prevention strategy should be adjusted according to the HPV characteristics of each group. HPV infection rates varied with seasons in Guizhou.

## Data Availability

All data generated or analyzed during this study are included in this published article.

## Abbreviation

HPV: Human papillomavirus

## References

[1] Martins TR, de Oliveira CM, Rosa LR (2016). HPV genotype distribution in Brazilian women with and without cervical lesions: correlation to cytological data[J]. Virol J.

[2] Chen Z, Jing Y, Wen Q (2017). E6 and E7 gene polymorphisms in human papillomavirus types-58 and 33 identified in Southwest China[J]. PLoS One.

[3] Scheinfeld N, Lehman DS (2006). An evidence-based review of medical and surgical treatments of genital warts[J]. Dermatology online journal.

[4] Muñoz N, Bosch FX, De Sanjosé S (2003). Epidemiologic classification of human papillomavirus types associated with cervical cancer[J]. N Engl J Med.

[5] Chen Z, Jing Y, Wen Q (2016). L1 and L2 gene polymorphisms in HPV-58 and HPV-33: implications for vaccine design and diagnosis[J]. Virol J.

[6] Castellsague X (2007). HPV and cervical Cancer in the world: 2007 report (section I continents and regions)[J]. Vaccine.

[7] Ursu RG, Onofriescu M, Nemescu D (2011). HPV prevalence and type distribution in women with or without cervical lesions in the northeast region of Romania[J]. Virol J.

[8] Chen Z, Wang Q, Ding X (2015). Characteristics of HPV prevalence in Sichuan Province, China[J]. Int J Gynecol Obstet.

[9] Zeng Z, Yang H, Li Z (2016). Prevalence and genotype distribution of HPV infection in China: analysis of 51,345 HPV genotyping results from China's largest CAP certified laboratory[J]. J Cancer.

[10] Zhao P, Liu S, Zhong Z (2018). Prevalence and genotype distribution of human papillomavirus infection among women in northeastern Guangdong Province of China[J]. BMC Infect Dis.

[11] Zhao R, Zhang WY, Wu MH (2009). Human papillomavirus infection in Beijing, People's Republic of China: a population-based study[J]. Br J Cancer.

[12] Chen X, Wallin KL, Duan M (2015). Prevalence and genotype distribution of cervical human papillomavirus (HPV) among women in urban Tianjin, China[J]. J Med Virol.

[13] Wang S, Wei H, Wang N (2012). The prevalence and role of human papillomavirus genotypes in primary cervical screening in the northeast of China[J]. BMC Cancer.

[14] Chen Q, Luo ZY, Lin M (2012). Prevalence and genotype distribution of human papillomavirus infections in women attending hospitals in Chaozhou of Guangdong province[J]. Asian Pac J Cancer Prev.

[15] Baboci L, Boscolo-Rizzo P, Holzinger D (2013). Evidence of the causal role of human papillomavirus type 58 in an oropharyngeal carcinoma[J]. Virol J.

[16] Anderson LA, O'Rorke MA, Wilson R (2016). HPV prevalence and type-distribution in cervical cancer and premalignant lesions of the cervix: a population-based study from Northern Ireland[J]. J Med Virol.

[17] Hibbitts S, Rieck GC, Hart K (2006). Human papillomavirus infection: an anonymous prevalence study in South Wales, UK[J]. Br J Cancer.

[18] Karanam B, Jagu S, Huh WK (2009). Developing vaccines against minor capsid antigen L2 to prevent papillomavirus infection[J]. Immunol Cell Biol.

